# Hierarchically Assembled Gigantic Fe/Co Cyanometallate Clusters Exhibiting Electron Transfer Behavior Above Room Temperature

**DOI:** 10.1002/advs.202402884

**Published:** 2024-06-14

**Authors:** Zi‐Yi Chen, Kai‐Ping Xie, Yue Cheng, Yi‐Fei Deng, Yuan‐Zhu Zhang

**Affiliations:** ^1^ Department of Chemistry Southern University of Science and Technology (SUSTech) Shenzhen 518055 China; ^2^ School of Chemistry and Materials Engineering Huizhou University Huizhou 516007 China

**Keywords:** cyanide‐bridged clusters, high‐nuclearity, metal‐to‐metal electron transfer, self‐assembly, versatile ligand

## Abstract

The construction of large and complex supramolecular architectures through self‐assembly is at the forefront of contemporary coordination chemistry. Notwithstanding great success in various systems using anionic bridges (e.g., O^2−^ or S^2−^) or organic ligands (e.g., pyridine or carboxylate ligands), the assembly of large cyanide‐bridged clusters with increasing nuclearity remains a formidable synthetic challenge. In this study, it is achieved in preparing two heterometallic cyanometallate clusters with unprecedented complexity, [Fe_20_Co_20_] (**1**) and [Fe_12_Co_15_] (**2**), by creating the “flexibility” through a versatile ligand of bis((1H‐imidazol‐4‐yl)methylene)hydrazine (H_2_L) and low‐coordinate cobalt. Complex **1** features a super‐square array of four cyanide‐bridged [Fe_4_Co_4_] cube subunits as the corners that are interconnected by four additional [FeCo] units, resulting in a torus‐shaped architecture. Complex **2** contains a lantern‐like core‐shell cluster with a triple‐helix kernel of [Co_3_L_3_] enveloped by a [Fe_12_Co_12_] shell. The combined structure analysis and mass spectrometry study reveal a hierarchical assembly mechanism, which sheds new light on constructing cyanometallate nanoclusters with atomic precision. Moreover, complex **1** undergoes a thermally induced electron‐transfer‐coupled spin transition (ETCST) between the diamagnetic {Fe^II^_LS_(µ‐CN)Co^III^_LS_} and paramagnetic {Fe^III^_LS_(µ‐CN)Co^II^_HS_} configurations (LS = low spin, HS = high spin) above room temperature, representing the largest molecule displaying electron transfer and spin transition characteristic.

## Graphical abstract

The largest electron‐transfer‐active molecule: Two unprecedentedly complex cyanometallate clusters, [Fe_20_Co_20_] (**1**) and [Fe_12_Co_15_] (**2**), which possess a super‐square and a lantern‐shaped core‐shell architecture, respectively, are synthesized through a self‐assembly process that utilizes archetype building blocks and a versatile N‐donor ligand. Complex **1**, in particular, exhibits a thermally induced electron‐transfer‐coupled spin transition (ETCST) above room temperature, earning it the distinction of being the largest electron‐transfer‐active molecule reported to date.

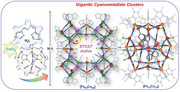

## 1 Introduction

The construction of large and complex supramolecular architectures through self‐assembly is at the forefront of contemporary coordination chemistry.^[^ ^1^, ^2^, ^3^, ^4^, ^5^ ^]^ The past decade has witnessed the prosperity of structurally fascinating molecular systems, including metal cycles,^[^ ^6^, ^7^, ^8^, ^9^, ^10^ ^]^ molecular knots,^[^ ^11^, ^12^, ^13^, ^14^ ^]^ metal‐organic polyhedra,^[^ ^15^, ^16^, ^17^ ^]^ and metallocages.^[^ ^18^, ^19^, ^20^, ^21^, ^22^, ^23^, ^24^ ^]^ Besides the aesthetic beauty, formidable synthetic challenges, and profound implications in nature, those compounds have already shown some promising applications including catalysis,^[^ ^25^, ^26^, ^27^ ^]^ separation,^[^ ^28^ ^]^ photoluminescence,^[^ ^29^ ^]^ and bio‐applications.^[^ ^30^, ^31^ ^]^ Taking advantage of their nanoscale size and elegant geometries, the intrinsic functionalities of their individual subcomponents such as luminescence, catalysis, and magnetic properties may be combined, integrated, or synergized, leading to the emergence of multi‐scale properties.^[^ ^32^, ^33^, ^34^, ^35^ ^]^

As one of the most prevalent bridging ligands, cyanide may transmit magnetic interaction and enable through‐bond electron transfer.^[^ ^36^, ^37^, ^38^, ^39^, ^40^, ^41^, ^42^, ^43^ ^]^ Based on the rigid linear linking mode of the cyanide bridges, the tailored building block techniques allow for the self‐assembly of small structural archetypes in a controlled manner, providing great potential for tailoring the magnetic properties through chemical design.^[^ ^44^ ^]^ One area that particularly benefits from this approach is the Fe/Co Prussian Blue analogs (PBAs), in which thermo‐ and/or photo‐induced electron‐transfer‐coupled spin transition (ETCST) between the diamagnetic {Fe^II^_LS_(µ‐CN)Co^III^_LS_} and paramagnetic {Fe^III^_LS_(µ‐CN)Co^II^_HS_} phases (LS = low spin, HS = high spin) has been systematically investigated.^[^ ^45^ ^]^ In addition to the prototype complexes like the [Fe_4_Co_4_] cubes,^[^ ^46^ ^]^ the [Fe_2_Co_2_] squares,^[^ ^47^ ^]^ as well as the [FeCo] dinuclear compounds,^[^ ^48^ ^]^ several compounds with more intricate structures and higher nuclearities have been synthesized and discovered to exhibit the ETCST behavior. The Oshio group, for instance, synthesized a tetradecanuclear [Fe_8_Co_6_] complex that features a twelve‐membered ring with alternate Fe and Co metal ions.^[^ ^49^ ^]^ They also reported an ETCST‐active [Fe_4_Co_6_] cage‐like cluster with a tetraethylammonium cation encapsulated within it.^[^ ^50^ ^]^

Notwithstanding the progress in harvesting cyanometallate complexes with predictable structures and tunable magnetic properties, constructing large cyanometallate clusters with increasing nuclearity remains a formidable synthetic challenge. In this vein, cyanide bridges prefer to bind just two metal centers, one at each end, to give a linear bridging arrangement, which provides some modicum of structural control.^[^ ^51^ ^]^ However, the rigid linear bridging mode is a major disadvantage for building high‐nuclearity clusters, as the assembly of large aggregates often requires certain “flexibility” of the anionic bridges (e.g., O^2−^ or S^2−^ in polyoxometalates or chalcogenides) or organic ligands.^[^ ^52^ ^]^ As such, only a handful of high‐nuclearity cyanide clusters with large ground spin states and enhanced magnetic functionalities have been reported. Pioneering works in this field were reported by the Long group, in which the large clusters of [Cr_12_Ni_12_] and [Cr_14_Ni_13_] were stabilized by the [(Me_3_tacn)Cr(CN)_3_]^−^ units and planar [Ni(CN)_4_]^2−^.^[^ ^53^, ^54^ ^]^ The Dunbar group obtained a [Mo(CN)_7_]‐based docosanuclear [Mo_8_Mn_14_] cyclic cluster, where the seriously bent Mn─N≡C─Mo cyanide linkages play an essential role in the formation of such a cyclic cluster.^[^ ^55^ ^]^ Later, the Sato group constructed an *O*‐symmetric [Fe_42_] cyanometallate cage, which contains both octahedral and square‐planar Fe ions, representing the largest cyanometallate cluster.^[^ ^56^ ^]^ And then its isostructural analogs [Fe_24_M_18_] (M = Fe, Ni, and Mn) were synthesized by the Liu group.^[^ ^57^ ^]^ These findings provided certain clues that the presence of a flexible linking node seems to be an indispensable ingredient in the formation of such high‐nuclearity cyanide‐bridged clusters.^[^ ^58^, ^59^ ^]^ More specifically, the introduction of building blocks or metal sites with low‐connection geometries (triangular, planar square, or tetrahedral) may provide enough room for the flexible cyanide linkages and facilitate the formation of nanoscale molecules.

**Scheme 1:**
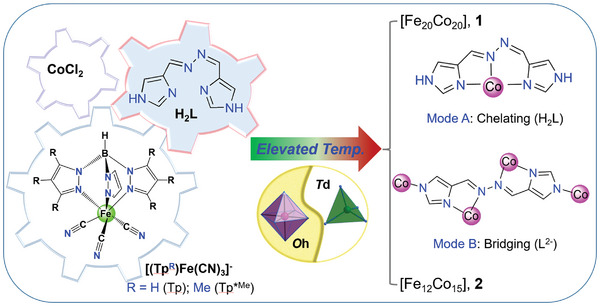
A rough synthetic route for assemblies of **1** and **2**, including the structures and coordination modes for the [(Tp^R^)Fe(CN)_3_]^−^ precursors, the 4‐ and 6‐coordinate cobalt, and H_2_L or its deprotonated form of L^2−^.

Recently, we have presented a synthetic strategy for the construction of cyanometallate clusters with increased nuclearity by introducing the 4‐coordinate cobalt under an elevated temperature.^[^ ^60^ ^]^ However, to meet the basic requirement for ETCST, N‐donor ancillary ligands need to be considered to achieve a full [CoN_6_] environment. We noticed that bis((1H‐imidazol‐4‐yl)methylene)hydrazine (H_2_L, **Scheme** **1**) is a highly versatile ligand with moderate ligand field. In specific, the ligand can function as a tridentate ligand and coordinates meridionally to the metal ion, as seen in both a mononuclear compound Fe(H_2_L)_2_(ClO_4_)_2_ and a 1D coordination polymer [Mn(H_2_L)(H_2_O)]_2_[Mo(CN)_7_]·6H_2_O.^[^ ^37^, ^61^ ^]^ When deprotonated, it can provide two additional vacant sites for linking to other metal ions, as observed in the fascinating Gyroidal metal‐organic frameworks of M^II^L (M = Zn, Mn, Cu, and Ni).^[^ ^62^ ^]^ It can also adapt a helix conformation, serving as a bis‐bidentate bridging ligand to bite two metal ions, in this manner, a dinuclear triple‐helicate compound Fe_2_(H_2_L)_3_(ClO_4_)_4_ was obtained.^[^ ^61^ ^]^ Herein, we report our success in exploring this ligand in the Fe/Co cyanometallate system with two obtained gigantic clusters: [{(Tp)Fe(CN)_3_}_20_{Co(H_2_L)}_8_{CoCl}_12_]·8MeOH·8MeCN·24H_2_O (**1**, Tp = hydridotris(pyrazol‐1‐yl)borate) and [Ph_3_PMe]_2_[{Co^II^_3_L_3_}{(Tp*^Me^)Fe(CN)_3_}_12_{CoCl}_12_]2Et_2_O·2H_2_O·2MeOH·3MeCN (**2**, Tp*^Me^ = hydridotris(3,4,5‐trimethylpyrazol‐1‐yl)‐borate). Complex **1** shows a super‐square array of four [Fe_4_Co_4_] cubes which are interlinked through four extra [FeCo] units, giving a torus‐shaped architecture. Complex **2** contains a lantern‐like core‐shell cluster, where a triple‐helix kernel of [Co^II^_3_L_3_] was enveloped by a [Fe_12_Co_12_] shell. We demonstrated the synergistic interaction of the ligand (H_2_L), iron cyanide building units, and tetrahedral/octahedral coordinate cobalt ions underlies the assembly of these complex cyanide‐bridged clusters. Furthermore, both structures strongly imply there is a supramolecular assembly process occurring, where small model complexes (dinuclears, square, cube) are condensed into their hierarchical aggregates. This hierarchical assembly mechanism is corroborated by the mass spectrometry study. Remarkably, complex **1** undergoes a thermally induced ETCST behavior above room temperature, which brings this intriguing phenomenon to the nanoscale.

## 2 Results and Discussion

### 2.1 General Remarks of the Synthesis and Characterization

Under an elevated temperature (≈50 °C), treating H_2_L, CoCl_2_, and [NBu_4_][(Tp)Fe(CN)_3_] in MeCN/MeOH (1:1) produced a cloudy dark‐green mixture, which was filtered and allowed to stand undisturbedly for slow evaporation. Dark‐green cubic crystals of **1** were obtained in a week, yielding: 49%. A similar reaction, involving [Ph_3_PMe][(Tp*^Me^)Fe(CN)_3_], CoCl_2_, and H_2_L in MeCN/MeOH (1:1) with the addition of excess NEt_3_, followed by vapor diffusion of Et_2_O, resulted in the dark‐red block‐like crystals of **2**, with a low yield of 15%. The addition of amines caused the ligand H_2_L to be deprotonated to L^2−^ in **2**. We hypothesized that the presence of numerous methyl groups on the Tp*^Me^ ligands contributed to the improved solubility of compound **2**. As a result, a viscous black oil was initially formed upon the diffusion of Et_2_O, which gradually turned into block‐like crystals after 2–3 weeks but in a low yield. Thermo‐gravimetric analysis (TGA) showed that both **1** and **2** desolvated easily above room temperature. The molecular skeleton of **1** began to decompose at nearly 200 °C, while that of **2** collapsed with the loss of lattice solvents (Figure S2, Supporting Information). The powder X‐ray diffraction (PXRD) pattern of **1** matched well with its single crystal X‐ray diffraction (SCXRD) simulation, confirming the purity of the bulk crystalline product (Figure S1, Supporting Information). However, no satisfactory PXRD data for **2** could be obtained due to its low crystallinity and possible decomposition.

### 2.2 Crystal Structure Description of Complex 1

**Figure 1:**
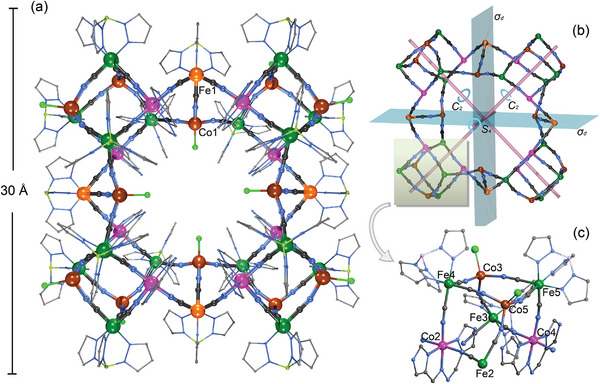
a) The molecular structure of the neutral super‐square cluster of [{(Tp)Fe^III^(CN)_3_}_4_{(Tp)Fe^II^(CN)_3_}_16_{Co^III^(H_2_L)}_8_{Co^II^Cl}_12_] in **1**, b) the [Fe_20_Co_20_(CN)_60_] molecular skeleton showing the super‐square structure in the *D_2d_* symmetry, c) the [Fe_4_Co_4_] subunit, formulated as {[(Tp)Fe^II^(CN)_3_]_4_[Co^III^(H_2_L)]_2_Co^II^_2_Cl_2_}, color code: Fe^III^ orange, Fe^II^ green, Co^III^ pink, Co^II^ brown, B lime green, Cl bright green, N blue, C gray.

Single crystal X‐ray diffraction (SCXRD) study at 100(2) K revealed that **1** crystallizes in the tetragonal space group of *P* $\bar{4}$2_1_ *c*. The structure of **1** features a torus‐like architecture with a neutral cyanide‐bridged 40‐metal core of [{(Tp)Fe(CN)_3_}_20_{Co(H_2_L)}_8_{CoCl}_12_] in the *D_2d_* symmetry (**Figure** **1**; Figures S4–S7, Supporting Information). The structure can be described as a “super‐square” consisting of four cubic [Fe_4_Co_4_] subunits at the corners, of which the neighboring cubes are inter‐connected through a [FeCo] pair. Two mirror planes defined by two Fe–CN–Co linkages of the opposite [FeCo] pairs are mutually perpendicular and intersect on a main axis of *S_4_*, which goes through the inversion center of the cluster. Thus, the asymmetric unit is only a quarter of the cluster and equally contains one [FeCo] pair of [{(Tp)Fe(CN)_3_}{CoCl}] (Fe1 and Co1) and one open cubic [Fe_4_Co_4_] subunit of [{(Tp)Fe(CN)_3_}_4_{Co(H_2_L)}_2_{CoCl}_2_] (Fe2 to Fe5, and Co2 to Co5).

Within the [Fe_4_Co_4_] cubic subunit, the iron and cobalt ions alternately reside in the corners, and two edges are disconnected (Co2···Fe3, Fe2···Co4), leading to a heavily distorted and open cube. Co3 and Co5 adopt a tetrahedral geometry (CoN_3_Cl), while Co2 and Co4 adopt an octahedral geometry each being chelated by one neutral H_2_L ligand in the *mer*‐tridentate coordination mode (Mode A, Scheme 1). Co2 is linked to two intra‐cube Fe atoms and another Fe atom of a side [FeCo] pair through the cyanide groups. Accordingly, each {(Tp)Fe(CN)_3_} unit of Fe4 and Fe5 links three intra‐cube Co centers via all three cyanide groups while each Fe2 or Fe3 unit links two intra‐cube Co centers and one intercube Co one. As such, each Fe_4_Co_4_ cube is double connected to two neighboring [FeCo] units, and vice versa. Part of the Fe─C≡N─Co cyanide linkages deviate significantly from linearity with selected Co─N≡C angles of 158(2)⁰ and 163(2)⁰ corroborating the heavy distortion of the cubes.

For the octahedral Co2 and Co4, the Co–N distances are quite short in the range of 1.88(2) to 1.93(3) Å, assigned to the low‐spin Co(III) ions, while for Co1, Co3, and Co5, the Co─N and Co─Cl bond lengths of 1.845(15) – 1.98(2) Å and 2.198(2) – 2.238(3) Å, respectively, are in good agreement with the high‐spin tetrahedral Co(II) ions. Based on the valence sum bond analysis and charge compensation, the formula of this cluster is thus expressed as [{(Tp)Fe^II^(CN)_3_}_16_{(Tp)Fe^III^(CN)_3_}_4_{Co^III^(H_2_L)}_8‐_{Co^II^Cl}_12_], in which only four Fe centers (Fe1 atoms) along the sides are in the trivalent low‐spin state while the majority ones (Fe2 to Fe5 in the cubes) are in the divalent low‐spin state.

### 2.3 Crystal Structure Description of Complex 2

**Figure 2:**
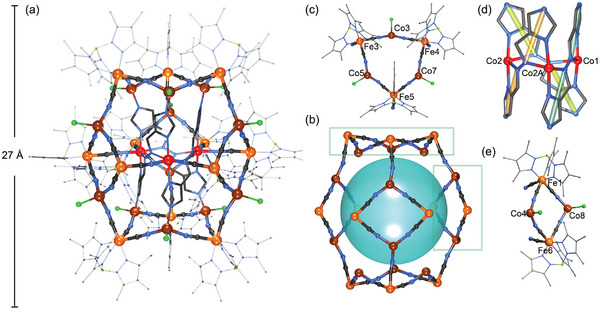
a) Perspective views the dianionic {[(Tp*^Me^)Fe(CN)_3_]_12_[Co(L)]_3_Co_12_Cl_12_} cluster in complex **2**, b) Co_12_Fe_12_(CN)_24_ shell structural unit, the Co_3_L_3_ inner cluster was indicated by a sky‐blue sphere, c) The [(Tp*^Me^)Fe(CN)_3_]_3_(CoCl)_3_ six‐membered ring structural unit, d) the triple‐helix Co_3_L_3_ core structural unit, e) [(Tp*^Me^)Fe(CN)_3_]_2_(CoCl)_2_ four‐member ring structural unit. Color code: Fe orange, Shell Co^II^ brown, Core Co^II^ red, B lime green, Cl bright green, N blue, C gray.

Single crystal X‐ray diffraction (SCXRD) study at 100(2) K revealed that **2** crystallizes in the monoclinic space group of *C*2/*c*. The structure of **2** features a dianionic lantern‐shaped cluster, [{Co_3_L_3_}@{(Tp*^Me^)Fe(CN)_3_}_12_{CoCl}_12_]^2−^ characterized by a trinuclear core of {Co_3_(L)_3_} encapsulated within a cyanide‐bridged 24‐metal shell containing twelve {(Tp*^Me^)Fe(CN)_3_} blocks and twelve {CoCl} units (**Figure** **2**; Figures S10 and S11, Supporting Information). The anionic cluster is charge‐balanced by two [P(Ph)_3_Me]^+^ cations. Although the molecular cluster appears to have a three‐fold rotational axis, crystallographically, it only exhibits a two‐fold (*C*_2_) one through Co1. Consequently, the asymmetric unit represents half of the cluster. Within the core, three Co atoms (Co1, Co2, and Co2A) are arranged into a planar triangular array, interconnected by three distinct L^2−^ ligands. Each L^2−^ ligand adopts the µ_4_‐η,^1^η,^2^η,^2^η1 coordination mode (Mode B, Scheme 1) to connect two inner (core) Co ions and two outer (shell) Co ions.

The 24‐nuclearity shell comprises two cyanide‐bridged crown‐like [Fe_3_Co_3_] hexagons (Figure 2c) as the bases and three cyanide‐bridged [Fe_2_Co_2_] squares (Figure 2e) as the side faces. Each hexagon links three squares through three cyanide groups, while each square links two hexagons through two cyanide groups, resulting in an elegant “hexagonal lantern”. It should be noted that the three twisted L^2−^ ligands, in conjunction with the bound inner Co atoms, form a triple‐helix trigonal prism, which provides two groups of outward N_imidazole_ donors in connection with the upper and bottom hexagon bases. Simultaneously, the core structure leaves six equatorial open sites to accept the inward cyanide groups from the shell. In other words, each Co···Co edge of the trigonal core is capped by a square subunit, giving rise to the elegant Co_3_L_3_@Co_12_Fe_12_ core‐shell structure.

Specifically, the Co ions within the core are situated in an octahedral geometry and each is coordinated to six N atoms, including two N_azo_, two N_imidazol_, and two N_cyanide_ atoms. The Co─N bond lengths vary in the range of 2.06(3) – 2.30(3) Å, indicating a divalent state. In addition, each Co ion of the shell, adopting a tetragonal geometry, is coordinated to three N_cyanide_ atoms and one terminal Cl atom. The Co─N and Co─Cl distances are in the range of 1.90(2) – 2.02(3) Å and 2.15(2) – 2.26(3) Å, respectively, which are consistent with those for a high‐spin Co(II) ion. Each {(Tp*^Me^)Fe(CN)_3_}^n−^ building block employs all three cyanide groups to link three Co atoms. The Fe─C and Fe─N bond lengths are in the range of 1.87(4)–2.09(4) and 1.90(3)–2.06(2) Å, respectively. Based on the valence sum bond analysis indicates that all Co centers are in the divalent high‐spin state, while the bond distances of the Fe centers in the current coordination sphere are not sensitive to the valences. However, two tri‐valent Fe ions are necessary according to the charge compensation, resulting in a formula of [{(Tp*^Me^)Fe^III^(CN)_3_}_10_{(Tp*^Me^)Fe^II^(CN)_3_}_2‐_{Co^II^Cl}_12_@{Co^II^_3_(L)_3_}]^2−^.

### 2.4 Room/Variable‐Temperature Infrared (IR) and ^57^Fe Mössbauer Spectra Study

**Figure 3:**
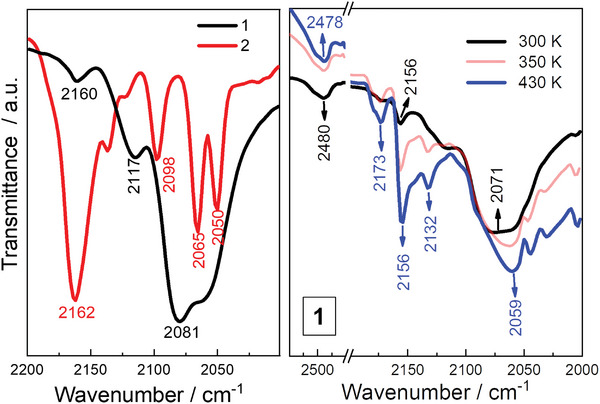
Room‐temperature solid‐state IR spectra of **1** and **2** (left) and variable‐temperature IR spectra of **1** (right) at a cooling rate of about 5 K min^−1^.

Room‐temperature infrared (IR) spectra data were collected for the solid samples **1** and **2** (293 K, **Figure** **3** and S3, Supporting Information). For **1**, the absorption at 2160 cm^−1^, assigned to the characteristic cyanide stretch (ν_CN_) for the [(Tp)Fe^III^(CN)_3_]^−^ units, is much weaker than those at ≈2081 cm^−1^ (with a shoulder at 2117 cm^−1^), which are corresponding to the cyanide stretches of the [(Tp)Fe^II^(CN)_3_]^2−^ units.^[^ ^63^ ^]^ This demonstrates that [(Tp)Fe^II^(CN)_3_]^2−^ units are dominant. For **2**, the cyanide stretches at 2162 cm^−1^ are significantly stronger than those between 2110–2050 cm^−1^ for the [(Tp)Fe^II^(CN)_3_]^2−^ units, in agreement with the crystallographic analysis of **2** for the existence of the [(Tp)Fe^II^(CN)_3_]^2−^ units, albeit in a small content. Given that the mixed‐valence Fe(II)‐Co(III) pairs within the cube subunits in **1** contain all‐nitrogen octahedral Co(III) ions, variable‐temperature IR spectra were further measured between 300 to 450 K to investigate the possible electron transfer behavior (Figure 3, right). Upon heating, the weak absorption band at 2156 cm^−1^ significantly grows in intensity with new absorptions appearing at 2173 and 2132 cm^−1^, and that of 2117 cm^−1^ disappearing. This clearly indicates the occurrence of an ETCST event from {Fe^II^_LS_‐CN‐Co^III^_LS_} to {Fe^III^_LS_‐CN‐Co^II^_HS_} in **1**.

**Figure 4:**
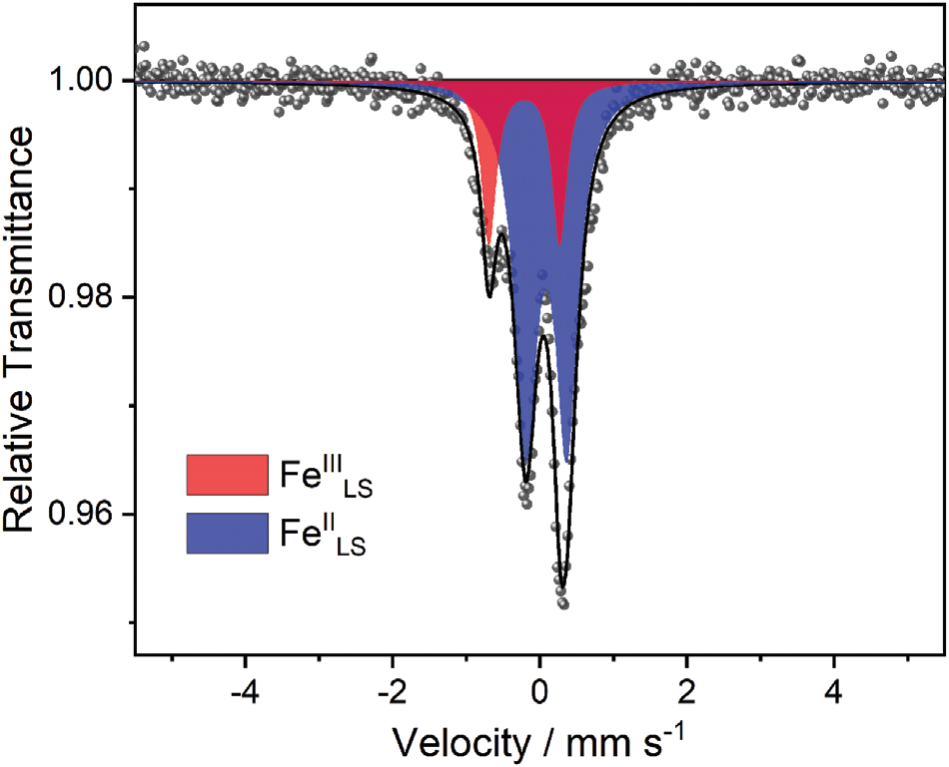
^57^Fe Mössbauer spectra of **1** at 100 K.

To gain insight into the mixed‐valance phase of **1**, ^57^Fe Mössbauer spectrum was collected at 100 K. As shown in **Figure** **4**, three unbalanced absorptions were observed. These absorptions could be well reproduced by two quadruple doublets with two sets of parameters, *δ* = −0.211 mm s^−1^, ΔE*_Q_* = 0.96 mm s^−1^, and *δ* = 0.088 mm s^−1^, ΔE_Q_ = 0.56 mm s^−1^, which could be assigned to Fe^III, LS^ and Fe^II, LS^, respectively.^[^ ^64^ ^]^ The peak area ratio for the two doublets was calculated as 23: 77, in good agreement with the 1:4 ratio of the Fe^III^ and Fe^II,^ deduced from the SCXRD analysis.

### 2.5 Magnetic Properties and Electron Transfer Behavior of Complex 1

**Figure 5:**
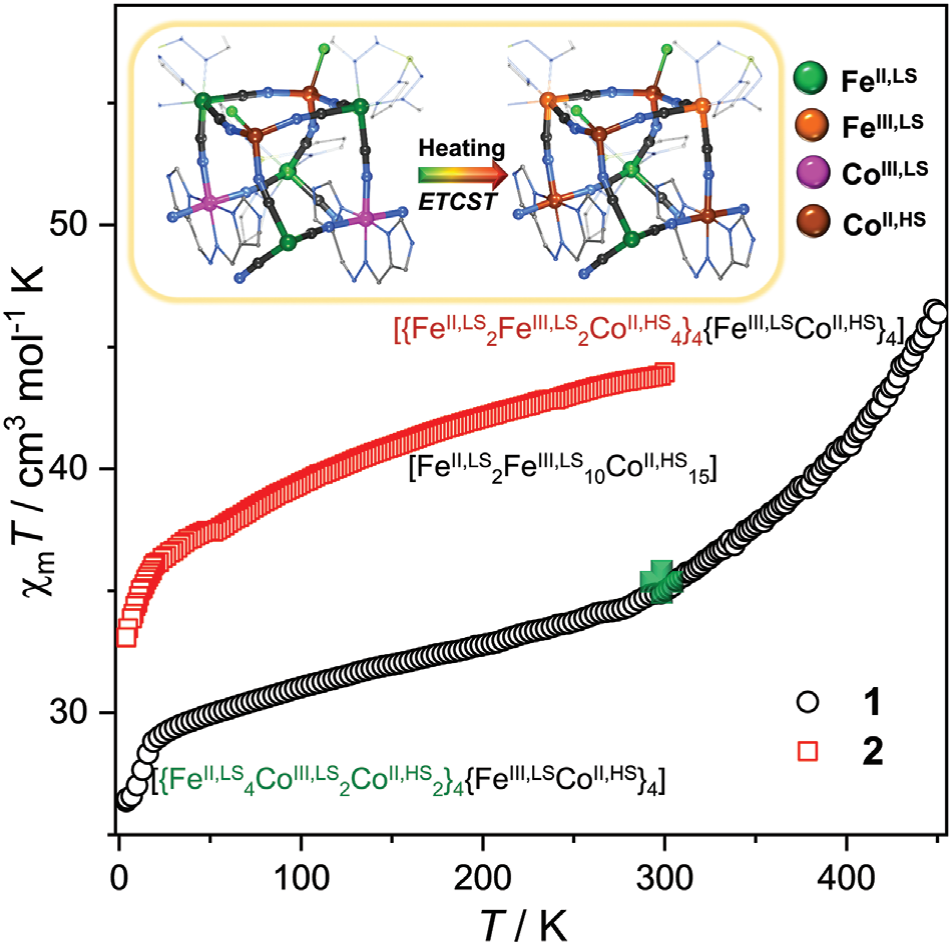
Temperature dependence of χ_m_ *T* obtained at 1 kOe for **1** and **2**, inset charge and spin‐state change of the [Fe_4_Co_4_] subunit in cluster **1** upon heating.

Variable‐temperature magnetic susceptibility data for **1** (2–450 K) and **2** (2–300 K) were collected under an applied direct‐current (dc) field of 1 kOe (**Figure** **5**). The χ_m_ *T* product at 300 K is 35.0 cm^3^ K mol^−1^ for **1**, which is consistent with the expected value for four LS Fe^III^ ions and twelve HS Co(II) ions (marked as [{Fe^II,LS^_4_Co^III,LS^_2_Co^II,HS^_2_}_4_{Fe^III,LS^Co^II,HS^}_4_]), whereas the low‐spin Fe(II) and Co(III) are diamagnetic with *S*_Fe(II)_ = 0 and *S*_Co(III)_ = 0. While the χ_m_ *T* product for **2** is 44.0 cm^3^ K mol^−1^ consisting with ten LS Fe(III) ions and fifteen HS Co(II) ions (marked as [Fe^II,LS^_2_Fe^III,LS^_10_Co^II, HS^_15_]). Assuming the LS Fe(III) ion had *S*_Fe(III)_ = 1/2 and *g*_Fe_ ≈ 2.5,^[^ ^13a^ ^]^ the HS Co(II) ions (*S*_Co(II)_ = 3/2) were estimated as *g*_Co_ ≈ 2.41 in **1** and *g*_Co_ ≈ 2.33 in **2**, respectively, indicating considerable orbital contributions. Upon cooling, both the χ_m_ *T* products decrease gradually before 20 K and then drop quickly, due to the possible intramolecular and intermolecular antiferromagnetic (AF) interactions, and zero‐field splitting effect. The *M*(*H*) curves measured at 2.0 K (Figure S13, Supporting Information) reveal that the magnetizations do not saturate even at 70 kOe, reaching maximum values of 28.2 and 32.5 *Nβ* for **1** and **2**, respectively. This indicates the presence of high magnetic anisotropy. Furthermore, the ac susceptibilities measured at zero dc field for both compounds do not exhibit any out‐of‐phase signals, thus ruling out the possibility of an SMM behavior (Figure S14, Supporting Information).

Notably, the χ_m_ *T* product of **1** increases dramatically upon heating from 300 to 450 K, reaching a value of 46.6 cm^3^ K mol^−1^at 450 K, which suggests nearly half of the {Fe^II,LS^Co^III,LS^} pairs are turned into {Fe^III,LS^Co^II,HS^} pairs. This result clearly confirms the occurrence of an ETCST event involving a transition between the diamagnetic {Fe^II^_LS_(µ‐CN)Co^III^_HS_} and paramagnetic {Fe^III^_LS_(µ‐CN)Co^II^_HS_} pairs within the cube subunits (Inset of Figure 5), as observed in the VT‐IR spectra study (vide supra).

### 2.6 Formation Mechanism of the Gigantic Clusters: Versatile Ligand, Four‐Coordinate Cobalt, and Hierarchical Assembly

The successful assembly of the first and second largest heterometallic cyanometallate clusters described in the title warrants further discussion. It is also worth noting that no such nuclearities and topologies were detected in other non‐cyanido‐bridged cluster complexes. First, the utilization of 1,2‐bis((1H‐imidazol‐4‐yl)methylene)hydrazine (H_2_L), a versatile ligand with multiple N‐donor sites, adjustable charge, and convertible geometry, is unprecedented. As a result, this ligand functions as a neutral *mer*‐tridentate ligand in **1**, outperforming other *mer*‐tridentate ligands (e.g., 2,2′:6′,2′′‐Terpyridine) due to its smaller size, leading to minimized steric effects and increased nuclearity. In **2**, the deprotonated ligand acts as a di‐topic bridge while also providing chelating sites. This unique coordination mode enables the encapsulation of the [Co_3_L_3_] moiety within a metal cyanide shell. Second, the introduction of negatively charged chloride and the elevated reaction temperature allows for the adoption of a tetrahedral geometry by part of the Co ions. This provides larger N–Co–N angles (105–110⁰) compared to octahedral cobalt, facilitating a more flexible connection and enough room for enlargement. Additionally, the negatively charged chloride ligands make the robust building blocks [Fe_2_Co_2_] neutral, promoting collisions between these species in solution.

To probe the possible formation path of the clusters, mass spectrometry measurements were conducted on the reaction solution of **1**. As illustrated in Figure S11 (Supporting Information), two *m*/*z* peaks were observed, corresponding to two molecular subunits. Specifically, a peak at 2093.56 *m/z* was attributed to [NaFe_4_Co_4_]^+^, while another peak at 927.66 *m/z* corresponded to [Na_2_Fe_2_Co_2_]^+^ (the molecular structures of these species are shown in the figure). The current MS results suggest that the [Fe_2_Co_2_] square is a prevalent species in the solution, which is consistent with previous findings in other Fe‐Co cyanide systems. Additionally, our data confirm that the assembly of this giant structure occurs through the condensation of smaller archetype clusters in the solution.

## 3 Conclusion

In summary, we synthesized two unprecedentedly complex cyanometallate clusters, [Fe_20_Co_20_] (**1**) and [Fe_12_Co_15_] (**2**), by incorporating the versatile N‐donor ligand of 1,2‐bis((1H‐imidazol‐4‐yl)methylene)hydrazine (H_2_L). Complex **1**, with a torus shape, features a super‐square array of cyanide‐bridged [Fe_4_Co_4_] cubes, while complex **2**, in a lantern shape, contains a core‐shell cluster with a triple‐helix kernel of [Co_3_L_3_] enveloped by a [Fe_12_Co_12_] shell. Notably, complex **1** undergoes a thermally induced ETCST involving the diamagnetic {Fe^II^_LS_(µ‐CN)Co^III^_LS_} and paramagnetic {Fe^III^_LS_(µ‐CN)Co^II^_HS_} pairs within the cubes, making it the largest cluster displaying this specific phenomenon. The flexible coordination modes of H_2_L with either, or not, deprotonation and the presence of 4‐coordinate cobalt ions due to the chloride ions were crucial for the increase in cluster size. This work paves the way for a sophisticated approach to construct nanoscale cyanometallate aggregates with atomic precision, enabling further exploration of their properties.

## 4 Experimental Section

### Materials

Warning! Although no problems were encountered in the experiments, cyanides were highly toxic. Especially under acid conditions. The materials should be handled in small quantities with great caution. The ligand 1,2‐bis((5H‐imidazol‐4‐yl)methylene)hydrazine (H_2_L)^[^ ^62^ ^]^ and the iron cyanide precursors (Bu_4_N)[(Tp)Fe(CN)_3_,^[^ ^65^ ^]^ (Ph_3_MeP)[(Tp*^Me^)Fe(CN)_3_]^[^ ^66^ ^]^ were prepared according to the reported literature. All chemicals and reagents were of analytical grade without further purification.

### Synthesis of Compound 1

A MeOH/MeCN (2.5 mL/2.5 mL) suspension of H_2_L (15.8 mg, 0.084 mmol) was allowed to stir at 55 ⁰C for 10 min. A dark‐red solution of CoCl_2_·6H_2_O (45.7 mg, 0.192 mmol) and (Bu_4_N)[(Tp)Fe(CN)_3_] (55.0 mg, 0.093 mmol) in MeOH‐MeCN (1.5 ml:1.5 ml) was added dropwise to the above solution under vigorous stirring. The resulting dark‐green mixture was heated at 55 ⁰C for about 30 min. After filtering, slow evaporation of the filtrate for about one week afforded dark‐green block cuboid crystals in a yield of 49.3% (based on Fe). Anal. Calc. for **1**: C_328_H_384_B_20_Cl_12_Co_20_Fe_20_N_232_O_32_: C, 35.98, H, 4.42, N, 26.17. Found: C, 36.09, H, 5.05, N, 26.48.

### Synthesis of Compound 2

H_2_L (23.4 mg, 0.124 mmol) and (Ph_3_MeP)[Tp*^Me^Fe(CN)_3_] (63.5 mg, 0.084 mmol) were dissolved into a 1:1 (v:v) mixture of MeOH/MeCN (3 mL) affording a red turbid solution, 35.0 µL Et_3_N was added to the above mixture, which was allowed to stir at 55 ⁰C for 10 min. A solution of CoCl_2_·6H_2_O (61.8 mg, 0.260 mmol) in MeOH‐MeCN (1.5mL:1.5 mL) was added dropwise to the above mixture. The resulting red mixture was heated at 55 ⁰C for 30 min before filtrating. Black red block‐like crystals of 2 were obtained in two weeks by vapor diffusion of Et_2_O into its filtrate to furnish a yield of about 15% (based on Fe). Anal. Calc. for **2**: B_12_C_330_H_416_Cl_12_Co_15_Fe_12_N_129_O_12_P_2_: C, 46.34, H, 4.90, N, 21.12. Found: C, 47.29, H, 5.13, N, 22.58.

### Basic Characterization

Infrared spectroscopy in the range 500–4000 cm^−1^ was measured on a Bruker VERTEX 80v FTIR spectrometer (ATR method). The powder X‐ray diffraction (PXRD) data of ground fine powder was collected on a Rigaku SmartLab powder X‐ray diffractometer with Cu Kα radiation (45 kV, 200 mA) between 5 and 50° (2*θ*). The thermo‐gravimetric analysis (TGA) was performed on a Mettler Toledo TGA‐2 thermal gravimetric analyzer. Mass spectrum measurement was conducted on a Thermo Scientific Q Exactive Hybrid Quadrupole‐Orbitrap Mass Spectrometer.

### Crystallography

The diffraction data for **1** and **2** were collected at 100 K on a Bruker D8 VENTURE diffractometer with Cu Kα (λ = 1.54718 Å) radiation. Lorentz/polarization corrections were applied during data reduction and the structures were solved by the direct method (SHELXT‐2014).^[^ ^67^ ^]^ Refinements were performed by full‐matrix least squares (SHELXL‐2014) on F^2^.^[^ ^68^ ^]^ Anisotropic thermal parameters were used for the non‐hydrogen atoms. Hydrogen atoms were added geometrically and refined using a riding model. The single crystal X‐ray characterization of **2** proved to be problematic. The crystals of compound **2** are quite fragile and weakly diffracting and will lose their crystallinity within seconds when leaving the mother liquid. A considerable number of restraints and constraints such as DFIX, DANG, SADI, FLAT, AFIX, and DELU were used to stabilize the refinement. Also, for this big cluster structure, significant “solvent voids” were present, and it is impossible to locate atoms therein because of very limited localized electron density features lying in the voids. The Squeeze function of the PLATON program was used to rule out the solvent problem. Data collection and structural refinement parameters are given in Table S1 (Supporting Information) and selected bond distances and angles are given in Table S2 and Table S3 (Supporting Information), CCDC‐2248475 (**1**) and CCDC‐2248820 (**2**) contain the crystallographic data that can be obtained via www.ccdc.cam.ac.uk/conts/retrieving.html (or from the Cambridge Crystallographic Data Centre, 12, Union Road, Cambridge CB21EZ, UK, fax: (+44) 1223‐336‐033, or deposit@ccdc.cam.ac.uk).

### ATR‐IR Experiments

Room‐temperature infrared spectroscopy in the range 500–4000 cm^−1^ was measured on a Bruker VERTEX 80v FTIR spectrometer (attenuated total reflection technique). The variable‐temperature IR spectra of **1** were measured at the same spectrometer using a Golden Gate High‐Temperature ATR accessory (Specac Company).

### Magnetic Measurements

Magnetic susceptibility data were measured on a SQUID (Superconducting Quantum Interference Device) Quantum Design MPMS3 magnetometer. For the *dc* magnetic measurement, the pristine crystals were isolated via filtration from the mother liquor and transferred into a plastic capsule. This capsule was introduced to the sample chamber using brass straw. Once the chamber was purged, the sample was quickly cooled to 100 K (30 K min^−1^) and ready for the subsequent measurements. Diamagnetic corrections were calculated from Pascal constants and applied to all the constituent atoms and sample holders.^[^ ^69^ ^]^

The synthesis, powder X‐ray diffraction pattern, thermo‐gravimetric curves, room temperature IR spectra, additional structure figures, ESI‐HRMS spectrum, field‐dependent magnetization data, crystallography, bond valence sum calculations of compounds **1** and **2**.

## Conflict of Interest

The authors declare no conflict of interest.

## Supporting information

Supporting Information

## Data Availability

The data that support the findings of this study are available from the corresponding author upon reasonable request.
