# Supplementary material for: Hierarchically Assembled Gigantic Fe/Co Cyanometallate Clusters Exhibiting Electron Transfer Behavior Above Room Temperature
Source: Adv Sci (Weinh). 2024 Jun 14;11(30):2402884. doi: 10.1002/advs.202402884 (PMC11321628; doi:10.1002/advs.202402884)
Supplement: Supplementary file 1 — Supporting Information [file ADVS-11-2402884-s001.docx]

Supporting Information

Hierarchically Assembled Gigantic Fe/Co Cyanometallate Clusters Exhibiting Electron Transfer Behavior Above Room Temperature

Zi-Yi Chen,‡ Kai-Ping Xie,‡ Yue Cheng, Yi-Fei Deng, and Yuan-Zhu Zhang*

Department of Chemistry, Southern University of Science and Technology, Shenzhen, Guangdong 518055, China

E-mail: *[zhangyz@sustech.edu.cn](mailto:zhangyz@sustech.edu.cn)*

**Table of Contents**

**General Experimental Section** • • • • • • • • • • • • • • • • • • • • • • • •**3**

**Additional Figures** • • • • • • • • • • • • • • • • • • • • • • • • • • •• • • • • **4**

**Crystallography**• • • • • • • • • • • • • • • • • • • • • • • • • •• • • • • • • • **13**

**References** • • • • • • • • • • • • • • • • • • • • • • • • • • • • • • • • • • • • • **18**

Experimental Procedures

Materials and Physical Techniques

Warning! Although no problems were encountered in our experiments, cyanides are highly toxic. Especially under acid conditions. The materials should be handled in small quantities with great caution.

The ligand 1,2-bis((5H-imidazol-4-yl)methylene)hydrazine (H_2_L)^1^ and the iron cyanide precursors (Bu_4_N)[(Tp)Fe(CN)_3_],^2^ (Ph_3_MeP)[(Tp*^Me^)Fe(CN)_3_]^3^ were prepared according to the reported literature. All chemicals and reagents were of analytical grade without further purification. Infrared spectroscopy in the range 500-4000 cm^−1^ was measured on a Bruker VERTEX 80v FTIR spectrometer (ATR method). The powder X-ray diffraction (PXRD) data of ground fine powder was collected on a Rigaku SmartLab powder X-ray diffractometer with Cu Kα radiation (45 kV, 200 mA) between 5 and 50° (2*θ*). Magnetic susceptibility data were measured on a SQUID MPMS3 magnetometer. Diamagnetic corrections were calculated from Pascal constants and applied to all the constituent atoms and sample holder.^4^ The thermos-gravimetric analysis (TGA) was performed on a Mettler Toledo TGA-2 thermal gravimetric analyzer. Mass spectrum measurement was conducted on a Thermo Scientific Q Exactive Hybrid Quadrupole-Orbitrap Mass Spectrometer.

Synthesis of [{(Tp)Fe(CN)_3_}_20_{Co(H_2_L)}_8_{CoCl}_12_]·8MeOH·8MeCN·24H_2_O (1)

A MeOH/MeCN (2.5 mL/2.5 mL) suspension of H_2_L (15.8 mg, 0.084 mmol) was allowed to stir at 55 ⁰C for 10 minutes. A dark-red solution of CoCl_2_·6H_2_O (45.7 mg, 0.192 mmol) and (Bu_4_N)[(Tp)Fe(CN)_3_] (55.0 mg, 0.093 mmol) in MeOH-MeCN (1.5ml:1.5ml) was added dropwise to the above solution under vigorous stirring. The resulting dark-green mixture was heated at 55 ⁰C for about 30 minutes. After filtering, slow evaporation of the filtrate for about one week afforded dark-green block cuboid crystals in a yield of 49.3% (based on Fe). Anal. Calc. for **1**: C_328_H_384_B_20_Cl_12_Co_20_Fe_20_N_232_O_32_: C, 35.98; H, 4.42; N, 26.17. Found: C, 36.09; H, 5.05; N, 26.48.

Synthesis of [Ph_3_PMe]_2_[{Co^II^_3_L_3_}{(Tp*^Me^)Fe(CN)_3_}_12_{CoCl}_12_]·2(Et_2_O)·2CH_3_OH·3CH_3_CN·2H_2_O (2)

H_2_L (23.4 mg, 0.124 mmol) and (Ph_3_MeP)[Tp*^Me^Fe(CN)_3_] (63.5 mg, 0.084 mmol) were dissolved into a 1:1 (v:v) mixture of MeOH/MeCN (3 mL) affording a red turbid solution, 35.0 µL Et_3_N was added to the above mixture, which was allowed to stir at 55 ⁰C for 10 minutes. A solution of CoCl_2_·6H_2_O (61.8 mg, 0.260 mmol) in MeOH-MeCN (1.5ml:1.5ml) was added dropwise to the above mixture. The resulting red mixture was heated at 55 ⁰C for 30 minutes before filtrating. Black red block-like crystals of **2** were obtained in two weeks by vapor diffusion of Et_2_O into its filtrate to furnish a yield of about 15% (based on Fe). Anal. Calc. for **2**: B_12_C_330_H_416_Cl_12_Co_15_Fe_12_N_129_O_12_P_2_: C, 46.34; H, 4.90; N, 21.12. Found: C, 47.29; H, 5.13; N, 22.58.

**Additional Figures**

**Figure S1.** Experimental powder X-ray diffraction pattern and simulated one from the single crystal XRD data of **1**.

**
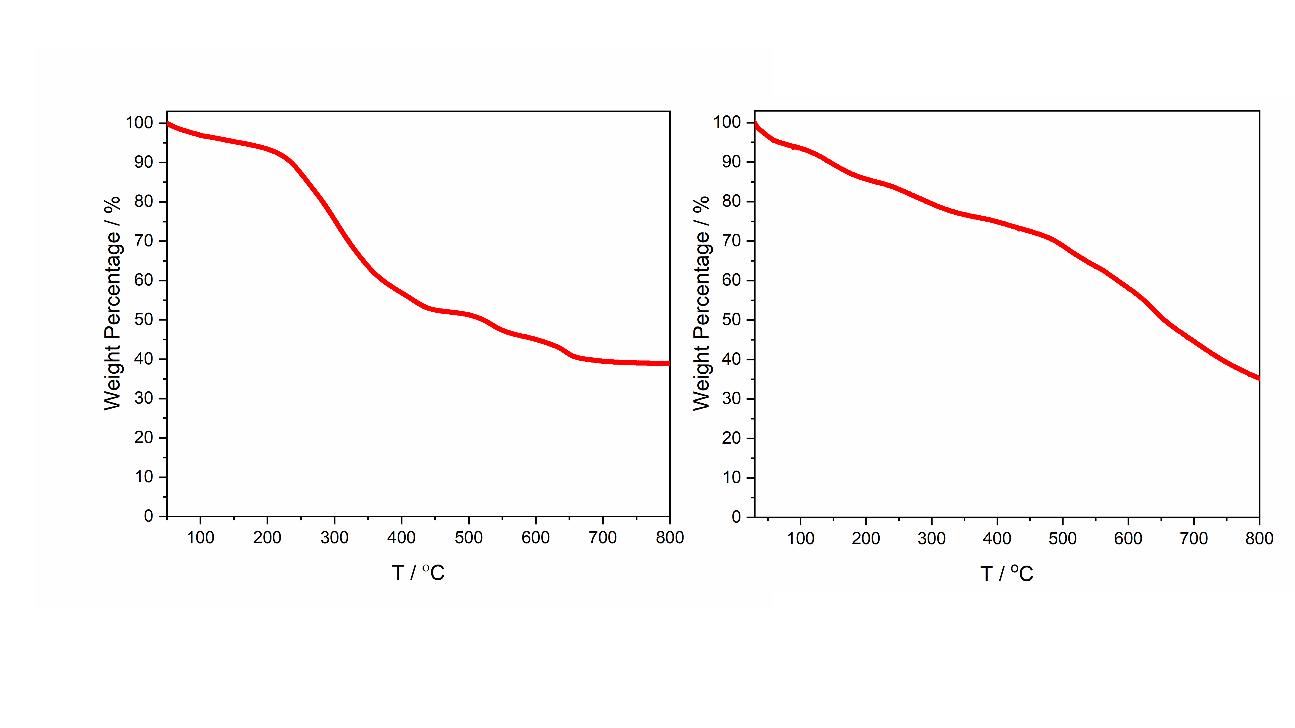
Figure S2.** Thermo-gravimetric curves of **1** (left) and **2** (right). Thermo-gravimetric analysis (TGA) showed that both **1** and **2** desolvated easily above room temperature. The molecular skeleton of **1** began to decompose at nearly 200°C, while that of **2** collapsed with the loss of lattice solvents


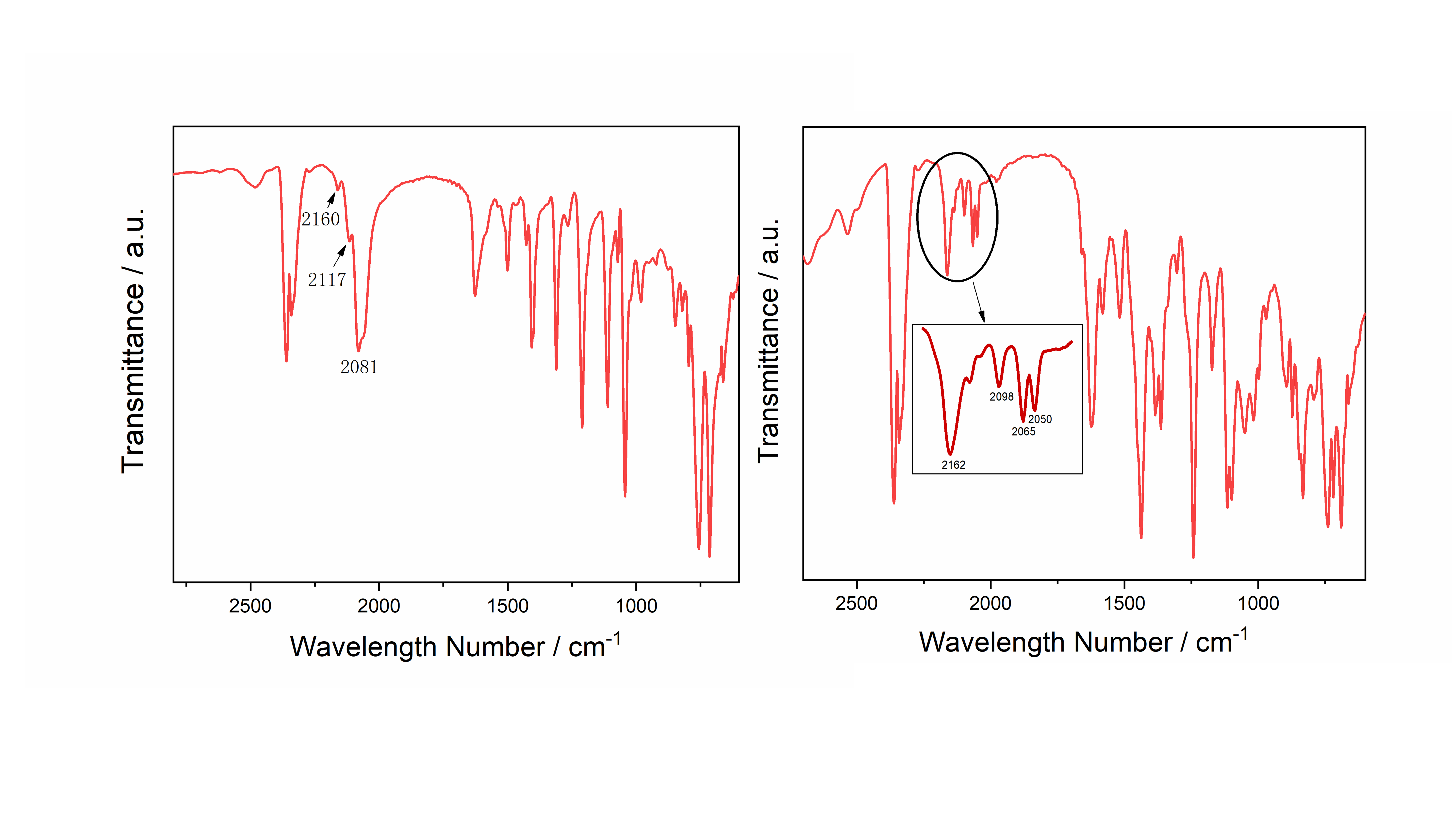


**Figure S3.** Room temperature IR spectra of **1** (left) and **2** (right).


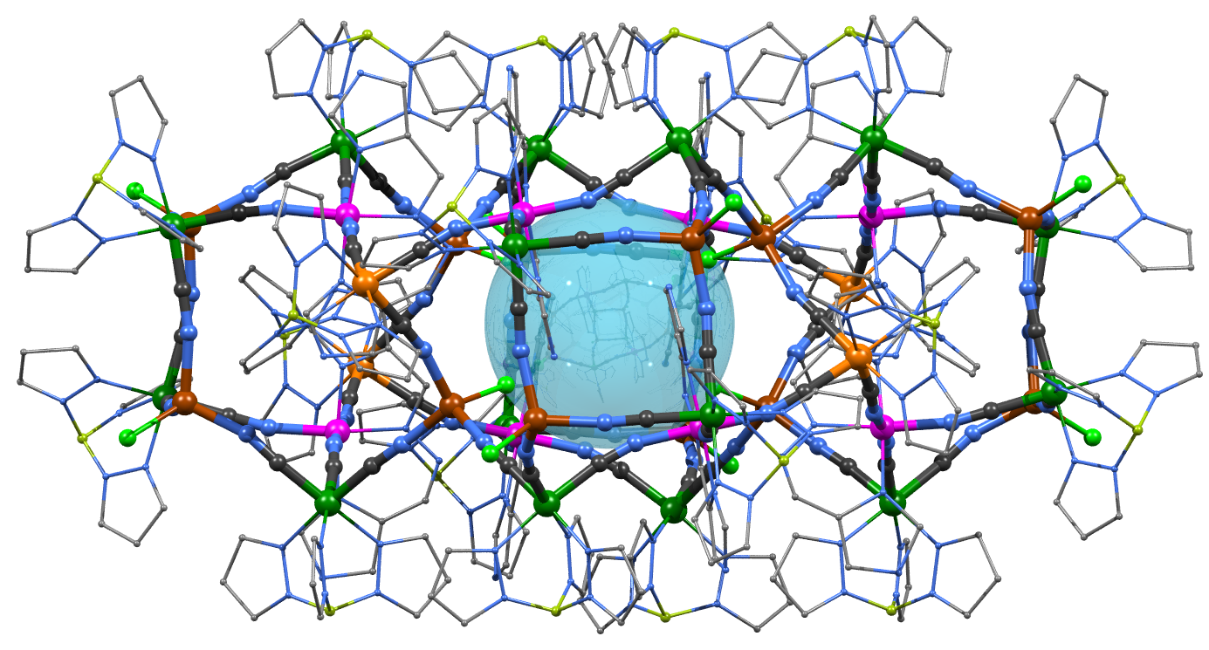


**Figure S4.** Side view of the neutral torus-shaped {[(Tp)Fe^III^(CN)_3_]_4_[(Tp)Fe^II^(CN)_3_]_16_[Co^III^(H_2_L)]_8_(Co^II^Cl_12_)_12_} cluster in complex **1**. The sky-blue ball is used to indicate the inner cavity of the cluster. Color code: Fe^III^ orange, Fe^II^ green, Co^III^ pink, Co^II^ brown, B lime green, Cl bright green, N blue, C gray.


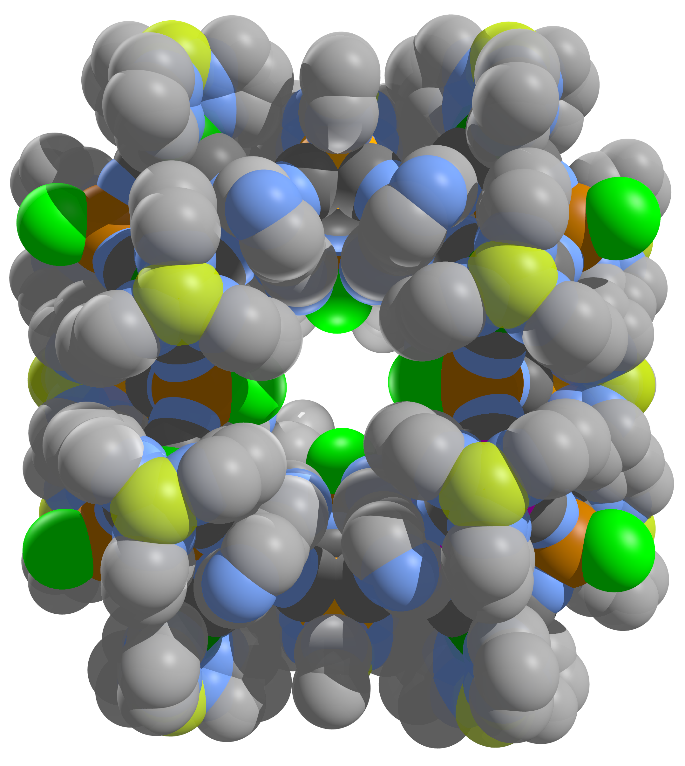


**Figure S5.** Space-Filling diagram of the {[(Tp)Fe^III^(CN)_3_]_4_[(Tp)Fe^II^(CN)_3_]_16_[Co^III^(H_2_L)]_8_(Co^II^Cl_12_)_12_} cluster in complex **1**. Color code: Fe^III^ orange, Fe^II^ green, Co^III^ pink, Co^II^ brown, B lime green, Cl bright green, N blue, C gray.


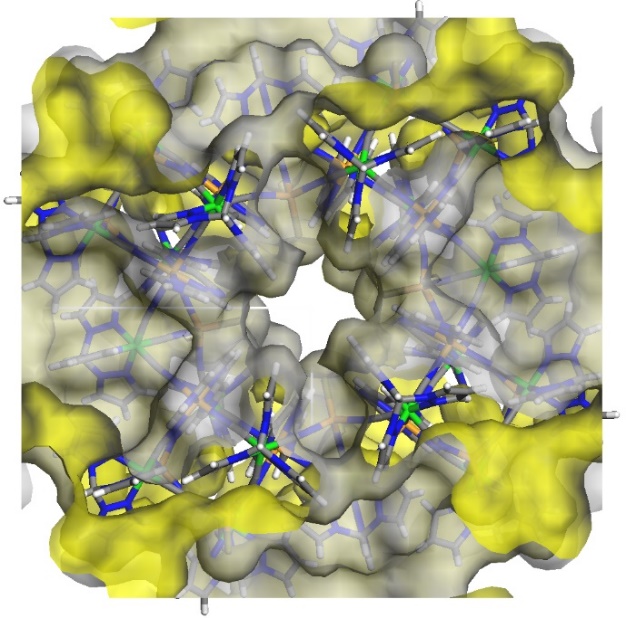

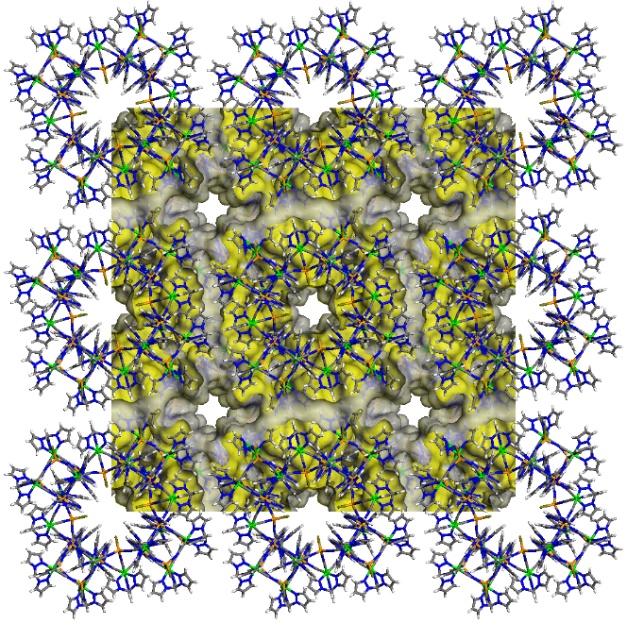


**Figure S6.** The pore surface structure of **1**.





**Figure S7.** SEM image of a crystal of compound **1**.


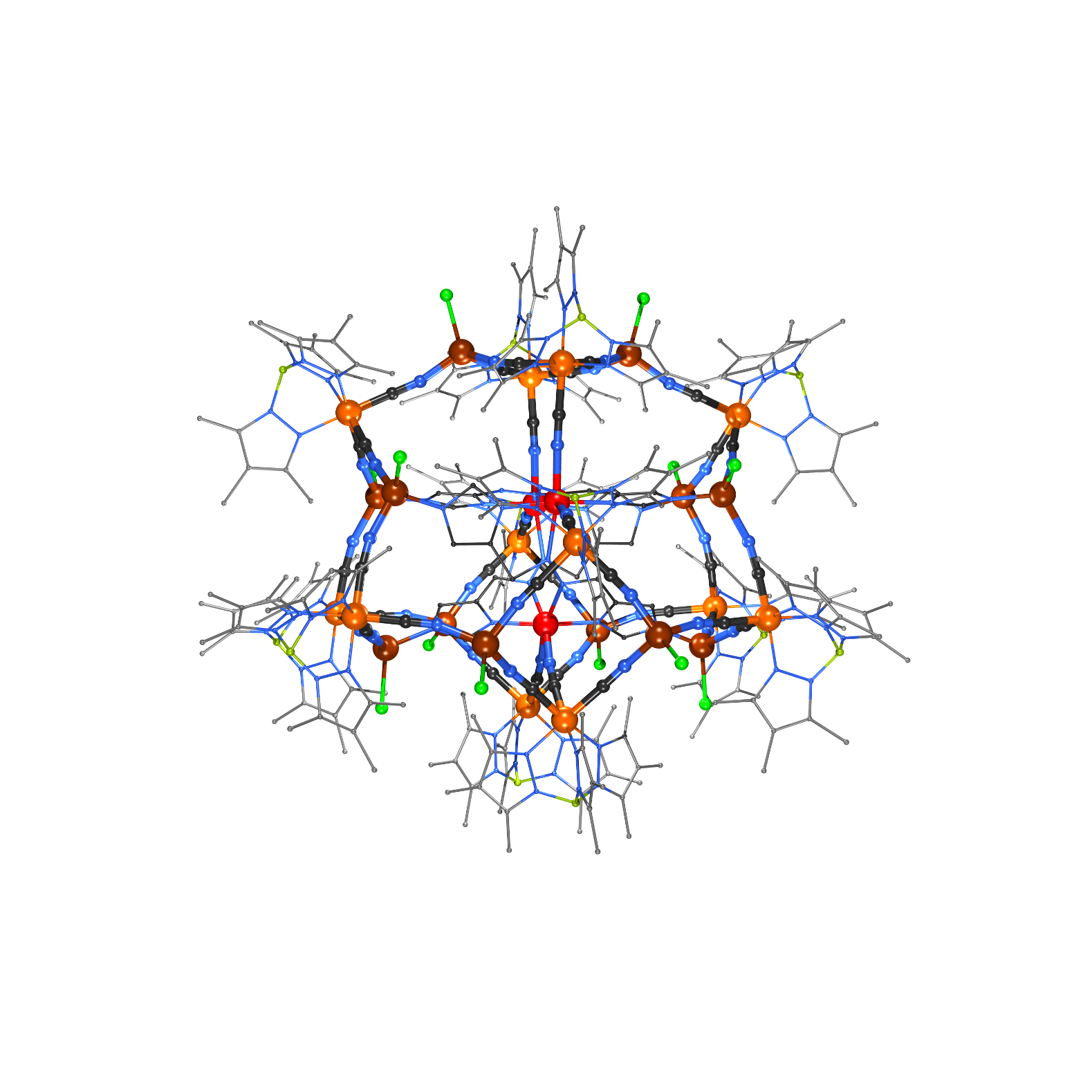


**Figure S8.** Side view of the dianionic {[(Tp*^Me^)Fe(CN)_3_]_12_[Co(L)]_3_Co_12_Cl_12_} cluster in complex **2**. Color code: Fe^III^ orange, Shell Co^II^ brown, Core Co^II^ red, B lime green, Cl bright green, N blue, C gray.


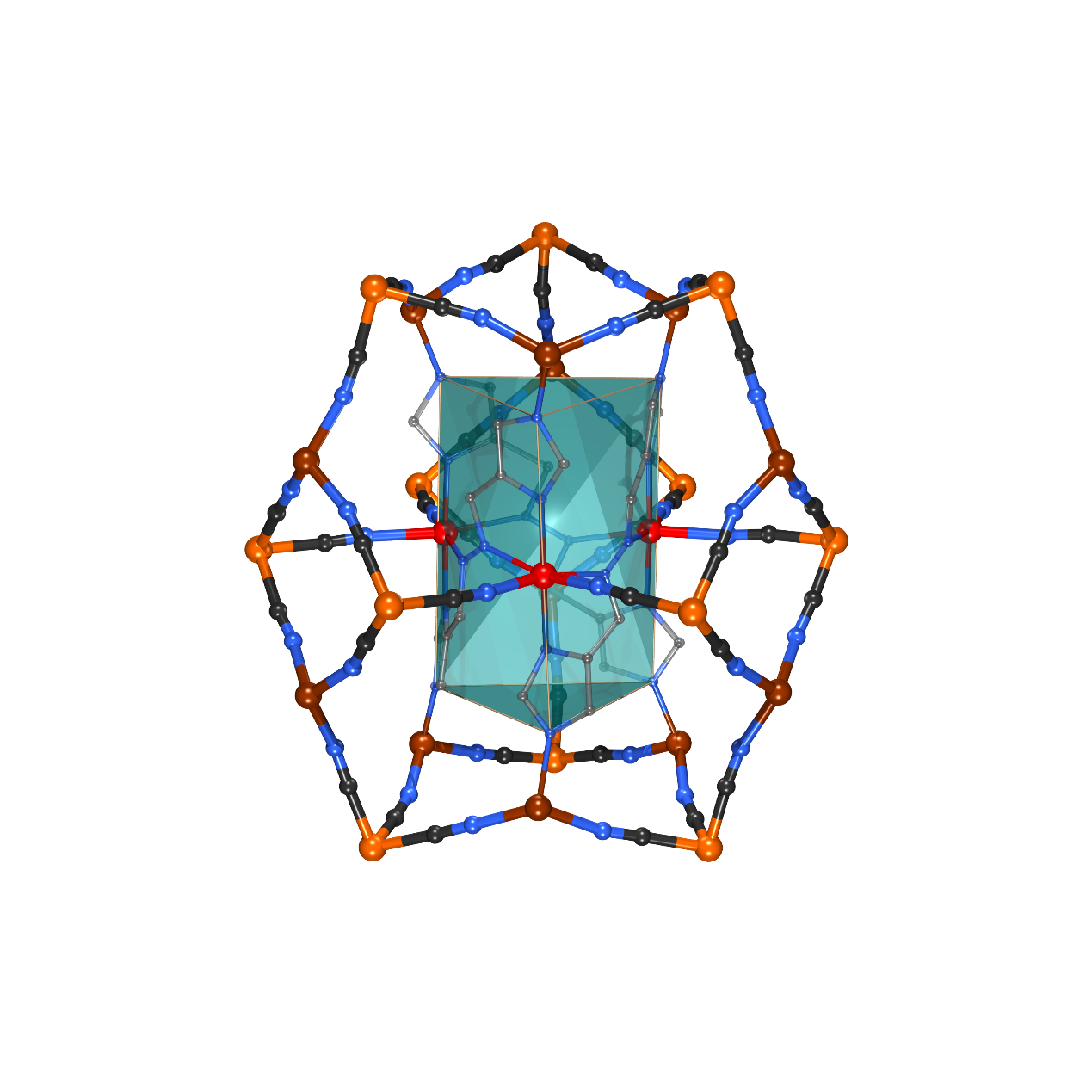


**Figure S9.** Co_3_L_3_@Co_12_Fe_12_(CN)_36_ structural unit. Color code: Fe^III^ orange, Shell Co^II^ brown, Core Co^II^ red.


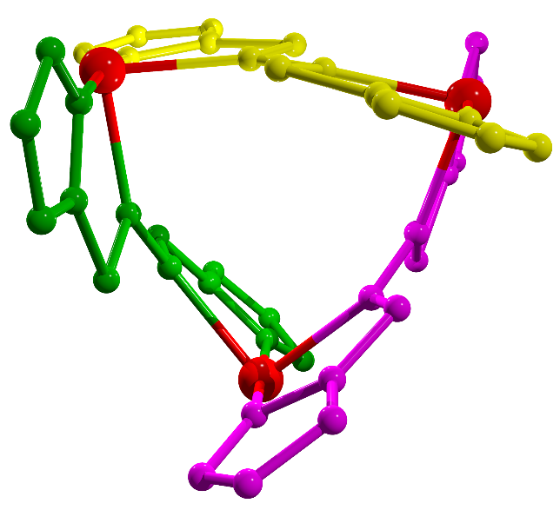


**Figure S10.** The helical conformation of the Co_3_L_3_ core in **2**. Three ligands are colored violet, green and yellow.


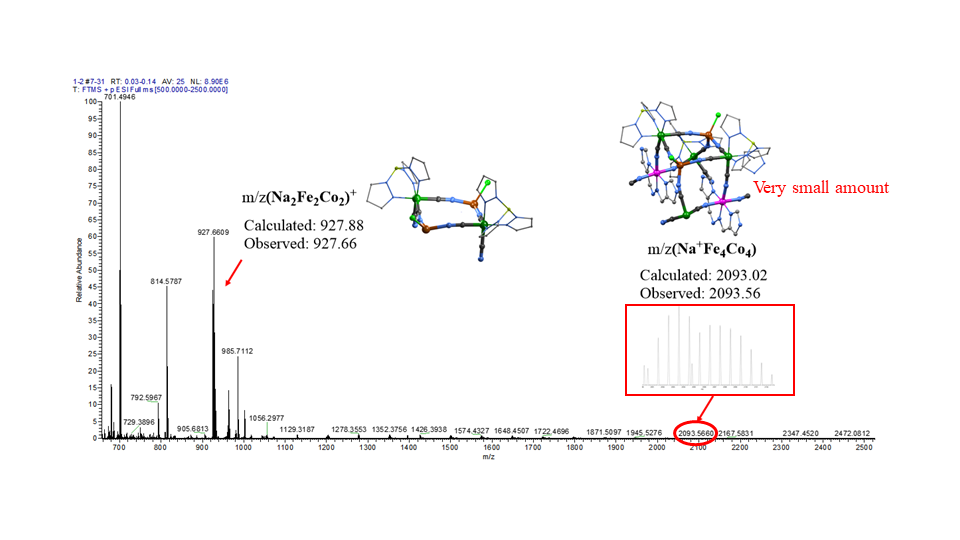


**Figure S11.** ESI-HRMS spectrum of the reaction mixture of **1** (positive mode) with identified peaks corresponding to the fragments of the cluster.





**Figure S12.** Temperature dependence of χ_m_*T* obtained at 1 kOe for **1**. Because of the loss of crystallinity and solvent upon heating, the χ_m_*T* curve in the cooling mode shows a more gradual ETSCT behavior at around 450-300K.








**Figure S13.** Field-dependent magnetization data of **1** (top) and **2** (bottom) at 2-5K.





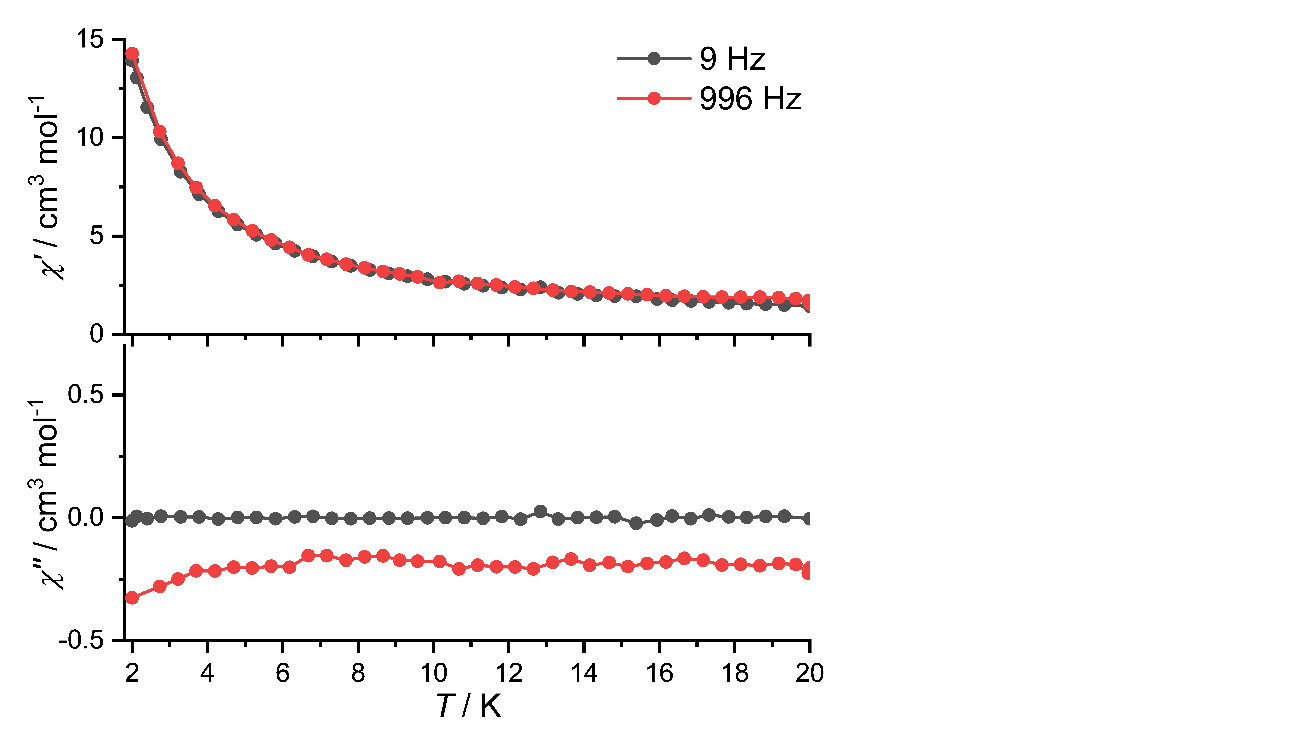


**Figure S14.** Alternating-current molar magnetic susceptibilities for **1** (top) and **2** (bottom) in zero dc field. No temperature dependence of the in-phase (χ′) and out-of-phase (χ″) parts was observed.

Crystallography

The diffraction data for **1** and **2** were collected at 100 K on a Bruker D8 VENTURE diffractometer with Cu Kα (*λ* = 1.54718 Å) radiation. Lorentz/polarization corrections were applied during data reduction and the structures were solved by the direct method (SHELXS-2014). Refinements were performed by full-matrix least squares (SHELXL-2014) on F^2^.^5^ Anisotropic thermal parameters were used for the non-hydrogen atoms. Hydrogen atoms were added geometrically and refined using a riding model. The single crystal X-ray characterization of **2** proved to be problematic. The crystals of compound **2** are quite fragile and weakly diffracting, and will lose its crystallinity within seconds when leaving the mother liquid. A considerable number of restrains and constrains such as DFIX, DANG, SADI, FLAT, AFIX and DELU were used to stable the refinement. Also for this big cluster structure, significant ‘solvent voids’ were present, and it is impossible to locate atoms therein because of very limited localized electron density features lying in the voids. Squeeze function of the PLATON program was used to rule out the solvent problem. Data collection and structural refinement parameters are given in Table S1 and selected bond distances and angles are given in Table S2 and Table S3, CCDC-2248475 (**1**) and CCDC-2248820 (**2**) contains the crystallographic data that can be obtained via www.ccdc.cam.ac.uk/conts/retrieving.html (or from the Cambridge Crystallographic Data Centre, 12, Union Road, Cambridge CB21EZ, UK; fax: (+44) 1223-336-033; or [deposit@ccdc.cam.ac.uk](mailto:deposit@ccdc.cam.ac.uk)).

**Table S1** Crystallographic data and structure refinement for complex **1** and **2**.

| Identification code | **1** | **2** |
| --- | --- | --- |
| Empirical formula | C_328_H_280_B_20_Cl_12_Co_20_Fe_20_N_236_O_32_ | B_12_C_330_Cl_12_Co_15_Fe_12_N_129_O_12_P_2_ |
| Formula weight | 10977.06 | 8101.80 |
| Temperature/K | 100.92 | 100.03 |
| Crystal system | Tetragonal | Monoclinic |
| Space group | *P*$\bar{4}$2_1_*c* | *C*2/*c* |
| *a*/Å | 31.4721(14) | 35.324(7) |
| *b*/Å | 31.4721(14) | 35.086(6) |
| *c*/Å | 34.972(2) | 36.732(7) |
| *α*/° | 90 | 90 |
| *β*/° | 90 | 96.957(5) |
| *γ*/° | 90 | 90 |
| Volume/Å^3^ | 34640(4) | 45190(14) |
| *Z* | 2 | 4 |
| *ρ*_calc_g/cm^3^ | 1.052 | 1.191 |
| *μ*/mm^‑1^ | 7.760 | 8.366 |
| F(000) | 11040.0 | 15960.0 |
| Crystal size/mm^3^ | 0.2 × 0.2 × 0.1 | 0.2 × 0.1× 0.1 |
| Radiation | CuKα (*λ* = 1.54178) | CuKα (*λ* = 1.54178) |
| 2θ range for data collection/° | 3.97 to 84.186 | 9.17 to73.164 |
| Index ranges | -27 ≤ *h* ≤ 27, -27 ≤ *k* ≤ 27, -30 ≤ *l* ≤ 30 | -27 ≤ *h* ≤ 27, -25 ≤ *k* ≤ 27, -26 ≤ *l* ≤ 28 |
| Reflections collected | 199043 | 33016 |
| Independent reflections | 11914 [*R*_int_ = 0.1081, *R*_sigma_ = 0.0479] | 10737 [*R*_int_ = 0.0727, *R*_sigma_ = 0.0967] |
| Data/restraints/parameters | 11914/1107/1448 | 10737/1732/2074 |
| Goodness-of-fit on F^2^ | 1.125 | 1.656 |
| Final R indexes [I>=2σ (I)] | *R*_1_ = 0.0999, *wR*_2_ = 0.2777 | *R*_1_ = 0.1532, *wR*_2_ = 0.4137 |
| Final R indexes [all data] | *R*_1_ = 0.1167, *wR*_2_ = 0.2962 | *R*_1_ = 0.1807, *wR*_2_ = 0.4401 |
| Largest diff. peak/hole / e Å^-3^ | 1.58/-0.70 | 0.80/-0.58 |
| Flack parameter | 0.024(4) |  |

**Table S2** Selected Bond lengths [Å] and angles [deg] for **1**.

| Fe1 |  | Fe2 |  |
| --- | --- | --- | --- |
| Fe1–N5 | 2.00(2) | Fe2–N4 | 2.07(3) |
| Fe1–N16 | 1.99(2) | Fe2–N13 | 2.02(2) |
| Fe1–N27 | 2.00(2) | Fe2–N17 | 1.98(2) |
| Fe1–C5 | 1.89(3) | Fe2–C13 | 1.83(3) |
| Fe1–C15 | 1.87(3) | Fe2–C23 | 1.90(4) |
| Fe1–C22 | 1.87(3) | Fe2–C30 | 1.81(3) |
| Fe3 |  | Fe4 |  |
| Fe3–N34 | 1.99(3) | Fe4–N28 | 1.97(2) |
| Fe3–N40 | 2.01(2) | Fe4–N30 | 2.01(3) |
| Fe3–N44 | 2.00(2) | Fe4–N36 | 1.99(2) |
| Fe3–C8 | 1.98(2) | Fe4–C11 | 1.86(3) |
| Fe3–C56 | 1.88(4) | Fe4–C19 | 1.83(3) |
| Fe3–C65 | 1.90(3) | Fe4–C37 | 1.91(3) |
| Fe5 |  | Co2 |  |
| Fe5–N12 | 2.00(3) | Co2–N7 | 1.91(3) |
| Fe5–N35 | 2.03(3) | Co2–N29 | 1.93(3) |
| Fe5–N41 | 2.01(3) | Co2–N43 | 1.87(3) |
| Fe5–C16 | 1.84(4) | Co2–N55 | 1.90(3) |
| Fe5–C35 | 1.81(4) | Co2–N56 | 1.92(2) |
| Fe5–C39 | 1.92(3) | Co2–N57 | 1.95(3) |
| Co1 |  | Co3 |  |
| Co1–Cl2 | 2.241(11) | Co3–Cl3 | 2.206(14) |
| Co1–N1 | 1.96(3) | Co3–N10 | 1.98(3) |
| Co1–N39 | 1.94(3) | Co3–N37 | 1.94(3) |
| Co1–N48^1^ | 2.00(3) | Co3–N47^2^ | 1.97(3) |
| Co4 |  | Co5 |  |
| Co4–N6 | 1.88(2) | Co5–Cl1 | 2.230(9) |
| Co4–N9 | 1.93(3) | Co5–N32 | 1.98(3) |
| Co4–N23^1^ | 1.89(3) | Co5–N38^2^ | 1.92(3) |
| Co4–N24 | 1.90(3) | Co5–N46 | 1.99(3) |
| Co4–N33 | 1.94(3) |  |  |
| Co4–N49 | 1.853(16) |  |  |
| Fe1 |  | Fe2 |  |
| C15–Fe1–N5 | 89.7(11) | C13–Fe2–N4 | 90.0(12) |
| C15–Fe1–N16 | 176.2(11) | C13–Fe2–N13 | 88.2(11) |
| C15–Fe1–N27 | 94.3(11) | C13–Fe2–N17 | 175.4(11) |
| C15–Fe1–C5 | 87.2(12) | C13–Fe2–C23 | 87.4(13) |
| C15–Fe1–C22 | 91.7(13) | C13–Fe2–C30 | 95.4(13) |
| Fe3 |  | Fe4 |  |
| C8–Fe3–N34 | 89.0(12) | C11–Fe4–N28 | 178.1(11) |
| C8–Fe3–N40 | 89.9(11) | C11–Fe4–N30 | 90.6(12) |
| C8–Fe3–N44 | 174.8(12) | C11–Fe4–N36 | 92.3(11) |
| C8–Fe3–C56 | 88.1(13) | C11–Fe4–C37 | 87.2(12) |
| C8–Fe3–C65 | 92.6(12) | C11–Fe4–C19 | 90.3(13) |
| Fe5 |  | Co2 |  |
| C35–Fe5–N12 | 177.4(12) | N7–Co2–N29 | 92.1(10) |
| C35–Fe5–N35 | 91.6(12) | N7–Co2–N56 | 91.7(10) |
| C35–Fe5–N41 | 90.1(13) | N7–Co2–N57 | 174.7(11) |
| C35–Fe5–C16 | 91.0(13) | N43–Co2–N7 | 89.4(10) |
| C35–Fe5–C39 | 90.8(13) | N55–Co2–N7 | 94.3(11) |
|  |  | Co2–N29≡C13 | 163(2) |
|  |  | Co2–N43≡C22 | 163(2) |
| Co1 |  | Co3 |  |
| N1–Co1–Cl2 | 112.7(8) | N10–Co3–Cl3 | 113.0(8) |
| N1–Co1–N48^1^ | 106.2(10) | N10–Co3–N47^2^ | 104.9(10) |
| N39–Co1–Cl2 | 109.2(8) | N37–Co3–Cl3 | 108.9(8) |
| N39–Co1–N1 | 109.4(10) | N37–Co3–N10 | 108.6(10) |
| N39–Co1–N48^1^ | 110.1(9) | N37–Co3–N47^2^ | 112.0(10) |
| Cl2–Co1–N48^1^ | 109.2(9) | Cl3–Co3–N47^2^ | 109.4(8) |
| Co1–N1≡C5 | 166(2) | Co3–N10≡C11 | 167(2) |
| Co1–N39≡C65 | 175(2) | Co3–N37≡C23 | 164(2) |
| Co1–N48^1^≡C30^1^ | 170(3) | Co3–N47^2^≡C35^2^ | 164(2) |
| Co4 |  | Co5 |  |
| N6–Co4–N19 | 92.1(11) | N32–Co5–Cl1 | 112.0(8) |
| N6–Co4–N23^1^ | 90.0(10) | N32–Co5–N46 | 111.8(10) |
| N6–Co4–N24 | 90.6(10) | N38^2^–Co5–Cl1 | 111.8(8) |
| N6–Co4–N33 | 82.7(11) | N38^2^–Co5–N32 | 104.9(10) |
| N49–Co4–N6 | 173.8(11) | N38^2^–Co5–N46 | 106.6(10) |
| Co4–N19≡C16 | 168(2) | Cl1–Co5–N46 | 109.5(7) |
| Co4–N23^1^≡C8^1^ | 160(2) | Co5–N32≡C19 | 165(2) |
| Co4–N24≡C15 | 161(2) | Co5–N38^2^≡C39^2^ | 170(2) |
|  |  | Co5–N46≡C56 | 158(2) |

^1^+Y,1-X,1-Z; ^2^1-Y,+X,1-Z

**Table S3** Selected Bond lengths [Å] and angles [deg] for **2**.

| Fe1 |  | Fe2 |  |
| --- | --- | --- | --- |
| Fe1–N2 | 2.00(2) | Fe2–N29 | 1.90(3) |
| Fe1–N6 | 1.98(3) | Fe2–C41 | 1.97(4) |
| Fe1–C33 | 1.96(4) | Fe2–N44 | 2.03(2) |
| Fe1–N41 | 1.98(2) | Fe2–C71 | 1.90(4) |
| Fe1–C81 | 1.98(5) | Fe2–N50 | 1.97(2) |
| Fe1–C102 | 1.86(4) | Fe2–C105 | 1.88(4) |
| Fe3 |  | Fe4 |  |
| Fe3–C10 | 1.81(5) | Fe4–C8 | 1.75(5) |
| Fe3–C30 | 1.85(4) | Fe4–C37 | 1.86(4) |
| Fe3–C35 | 1.89(4) | Fe4–C42 | 1.85(4) |
| Fe3–N40 | 2.04(2) | Fe4–N18 | 1.97(2) |
| Fe3–N57 | 2.05(2) | Fe4–N19 | 1.98(2) |
| Fe3–N62 | 1.97(2) | Fe4–N45 | 2.02(2) |
| Fe5 |  | Fe6 |  |
| Fe5–C9 | 1.87(4) | Fe6–C29 | 1.93(4) |
| Fe5–C96 | 1.87(5) | Fe6–C75 | 1.88(5) |
| Fe5–C141 | 1.83(5) | Fe6–C95 | 2.07(5) |
| Fe5–N22 | 1.97(2) | Fe6–N48 | 1.96(3) |
| Fe5–N25 | 2.05(2) | Fe6–N59 | 1.96(3) |
| Fe5–N52 | 1.98(2) | Fe6–N65 | 1.93(3) |
| Co1 |  | Co2 |  |
| Co1–N8^1^ | 2.06(2) | Co2–N1 | 2.18(3) |
| Co1–N8 | 2.06(2) | Co2–N10 | 2.16(3) |
| Co1–N12 | 2.08(3) | Co2–N15 | 2.25(3) |
| Co1–N12^1^ | 2.08(3) | Co2–N32^1^ | 2.06(10) |
| Co1–N31 | 2.28(3) | Co2–N37 | 2.059(19) |
| Co1–N31^1^ | 2.28(3) | Co2–N60 | 2.25(4) |
| Co3 |  | Co4 |  |
| Co3–Cl1 | 2.284(11) | Co4–Cl2 | 2.245(11) |
| Co3–N9^1^ | 2.00(6) | Co4–N7 | 1.90(3) |
| Co3–N16 | 1.98(3) | Co4–N11 | 1.88(3) |
| Co3–N23 | 1.96(3) | Co4–N27 | 1.97(3) |
| Co5 |  | Co6 |  |
| Co5–Cl3 | 2.261(13) | Co6–Cl5 | 2.239(14) |
| Co5–N36 | 1.962(18) | Co6–N4^1^ | 1.93(4) |
| Co5–N54 | 1.98(3) | Co6–N14 | 1.95(4) |
| Co5–N61 | 1.96(3) | Co6–N35 | 1.92(4) |
| Co7 |  | Co8 |  |
| Co7–Cl4 | 2.235(12) | Co8–Cl6 | 2.218(13) |
| Co7–N13 | 1.94(4) | Co8–N5^1^ | 1.94(3) |
| Co7–N24 | 2.02(4) | Co8–N34 | 1.94(3) |
| Co7–N33 | 1.98(2) | Co8–N38^1^ | 1.93(4) |
| Fe1 |  | Fe2 |  |
| C33–Fe1–N2 | 91.1(11) | C71–Fe2–C105 | 88.6(15) |
| C33–Fe1–N6 | 176.9(14) | C71–Fe2–N29 | 90.7(14) |
| C33–Fe1–N41 | 93.3(12) | C71–Fe2–C41 | 84.1(15) |
| C33–Fe1–C81 | 88.9(15) | C71–Fe2–N44 | 174.7(14) |
| C33–Fe1–C102 | 85.2(14) | C71–Fe2–N50 | 94.2(13) |
| Fe3 |  | Fe4 |  |
| C30–Fe3–N40 | 89.6(13) | C8–Fe4–C37 | 86.3(17) |
| C30–Fe3–N57 | 92.9(13) | C8–Fe4–N19 | 91.7(15) |
| C30–Fe3–N62 | 178.3(13) | C8–Fe4–C42 | 89.4(17) |
| C30–Fe3–C35 | 91.2(15) | C8–Fe4–N18 | 177.4(15) |
| C30–Fe3–C10 | 89.5(16) | C8–Fe4–N45 | 93.5(16) |
| Fe5 |  | Fe6 |  |
| C9–Fe5–N22 | 89.0(13) | C29–Fe6–C95 | 90.0(16) |
| C9–Fe5–N25 | 86.8(14) | C29–Fe6–N59 | 89.7(15) |
| C9–Fe5–N52 | 173.9(14) | C29–Fe6–C75 | 85.4(16) |
| C9–Fe5–C96 | 91.2(16) | C29–Fe6–N48 | 178.2(15) |
| C9–Fe5–C141 | 93.8(19) | C29–Fe6–N65 | 96.7(15) |
| Co1 |  | Co2 |  |
| N8–Co1–N8^1^ | 175.1(13) | N1–Co2–N15 | 92.2(10) |
| N8–Co1–N12 | 93.0(9) | N1–Co2–N60 | 169.6(12) |
| N8–Co1–N12^1^ | 90.4(10) | N1–Co2–N10 | 94.0(10) |
| N8–Co1–N31^1^ | 99.8(12) | N1–Co2–N32^1^ | 92(3) |
| N8–Co1–N31 | 76.7(12) | N1–Co2–N37 | 89.3(10) |
| Co1–N12≡C33 | 175(2) | Co2–N1≡C29 | 175(3) |
|  |  | Co2–N10≡C71 | 174(3) |
| Co3 |  | Co4 |  |
| N9^1^–Co3–Cl1 | 115(3) | N7–Co4–Cl2 | 114.2(12) |
| N16–Co3–Cl1 | 107.1(10) | N7–Co4–N27 | 105.0(13) |
| N16–Co3–N9^1^ | 98(4) | N11–Co4–N7 | 108.1(14) |
| N23–Co3–Cl1 | 102.8(9) | N11–Co4–Cl2 | 109.5(8) |
| N23–Co3–N9^1^ | 111(4) | N27–Co4–N11 | 118.2(12) |
| N23–Co3–N16 | 123.3(12) | N27–Co4–Cl2 | 102.1(8) |
| Co3–N16≡C37 | 167(3) | Co4–N7≡C95 | 170(5) |
| Co3–N23≡C30 | 180(4) | Co4–N11≡C35 | 164(3) |
|  |  | Co4–N27≡C102 | 160(3) |
| Co5 |  | Co6 |  |
| N54–Co5–Cl3 | 110.5(9) | N4^1^–Co6–Cl5 | 110.8(11) |
| N61–Co5–Cl3 | 109.5(10) | N4^1^–Co6–N14 | 101.7(13) |
| N36–Co5–N54 | 103.3(12) | N14–Co6–N35 | 109.2(13) |
| N36–Co5–Cl3 | 109.5(8) | N14–Co6–Cl5 | 112.3(10) |
| N36–Co5–N61 | 106.1(12) | N4^1^–Co6–N35 | 111.8(13) |
| N61–Co5–N54 | 117.5(13) | N35–Co6–Cl5 | 110.7(10) |
| Co5–N54≡C10 | 166(3) | Co6–N14≡C105 | 176(3) |
| Co5–N61≡C9 | 170(3) | Co6–N35≡C141^1^ | 167(4) |
|  |  | Co6–N4^1^≡C41^1^ | 167(4) |
| Co7 |  | Co8 |  |
| N24–Co7–Cl4 | 110.3(9) | N5^1^–Co8–Cl6 | 111.5(10) |
| N13–Co7–Cl4 | 108.3(10) | N5^1^–Co8–N34 | 105.5(13) |
| N13–Co7–N24 | 115.0(13) | N34–Co8–N38^1^ | 119.7(13) |
| N33–Co7–Cl4 | 111.7(8) | N34–Co8–Cl6 | 111.4(8) |
| N13–Co7–N33 | 97.5(13) | N5^1^–Co8–N38^1^ | 106.8(14) |
| N33–Co7–N24 | 113.3(11) | N38^1^–Co8–Cl6 | 102.0(11) |
| Co7–N13≡C96 | 168(4) | Co8–N5^1^≡C81^1^ | 166(3) |
| Co7–N24≡C8 | 164(3) | Co8–N34≡C42 | 165(3) |
|  |  | Co8–N38^1^≡C75^1^ | 167(4) |

^1^1-X,+Y,3/2-Z

**Table S4** Bond valence sum calculations for Co atoms of **1** and **2**.

| **1** | Co1(II, HS) | Co2(III, LS) | Co3(II, HS) | Co4(III, LS) | Co5(II, HS) |
| --- | --- | --- | --- | --- | --- |
|  | 2.11 | 3.03 | 2.18 | 3.18 | 2.15 |
| **2** | Co1(II, HS) | Co2(II, HS) | Co3(II, HS) | Co4 (II, HS) | Co5 (II, HS) |
|  | 1.99 | 1.86 | 1.99 | 2.33 | 2.09 |
|  | Co6(II, HS) | Co7(II, HS) | Co8(II, HS) |  |  |
|  | 2.26 | 2.07 | 2.28 |  |  |

Bond Valence = exp((*R_0_*-*R*)/*B*).^6^

**References**

(1) Zhou, X.-P.; Liu, M.; Li, J.; Li, D.; *J. Am. Chem. Soc.* **2012**, *134*, 67-70.

(2) Lescouëzec, R.; Vaissermann, J.; Lloret, F.; Julve, M.; Verdaguer, M. *Inorg. Chem*. **2002**, *41*, 5943−5945.

(3) Zheng, C.; Xu, J.; Wang, F.; Tao, J.; Li, D. *Dalton Trans.* **2016**, *45*, 17254–17263.

(4) Carlin, R. L. in *Magnetochemistry*. Springer-Verlag Press: Berlin, Heidelbeg, **1986**.

(5)(a) Sheldrick, G. M. in *SHELXT-2014, Program for the solution of crystal structures*, University of Göttingen, Göttingen, Germany, **2014**; (b) Sheldrick, G. M. in *SHELXL-2014, Program for Crystal Structure Refinement*, University of Göttingen, Göttingen, Germany, **2014**.

(6) Brown, I. D. Bond Valence Parameters. http://www.iucr.org/resources/data/datasets/bondvalence-parameters.
